# Minimizing the Risk of Perioperative Bleeding in a Child with Hemophilia A during Dental Rehabilitation under General Anesthesia: A Case Report

**DOI:** 10.5005/jp-journals-10005-1223

**Published:** 2013-10-14

**Authors:** Hisham Yehia El Batawi

**Affiliations:** Assistant Professor, Department of Pediatric Dentistry, Sharjah University, Sharjah, United Arab Emirates, Phone: 971508970564 e-mail: helbatawi@sharjah.ac.ae

**Keywords:** Child, Dental rehabilitation, General anesthesia, Hemophilia, Bleeding

## Abstract

Hemophilia, among other bleeding disorders, raises concerns for dental service providers who routinely use sharp hand and rotary instruments, address highly vascular soft tissue and provide dental extractions. In pediatric dentistry, dealing with fearful or irritable children increases the possibility of trauma and subsequent bleeding risks in hemophilic pediatric dental patients. In the current report, we discuss how anesthetic, pediatric and dental management may contribute to the delivery of safe and complete dental treatment for such children. This report describes the safe performance of dental treatments, including multiple extractions under general anesthesia, in a hemophilic child.

**How to cite this article:** El Batawi HY. Minimizing the Risk of Perioperative Bleeding in a Child with Hemophilia A during Dental Rehabilitation under General Anesthesia: A Case Report. Int J Clin Pediatr Dent 2013;6(3):217-222.

## INTRODUCTION

Hemophilia is a group of disorders of hemostasis that result from deficiency of blood coagulation factors. Hemophilia is an inherited X chromosome-linked bleeding disorder that affects approximately one in 5000 to 7500 males.^1^ In Saudi Arabia, where this case was managed, parental consanguinity contributes to the prevalence of hereditary diseases, such as hemophilia. Unfortunately, no local incidence reports of hemophilia are available in Saudi Arabia.^2^

### Types of Hemophilia

Hemophilia A (Online Mendelian Inheritance in Man (OMIM) number 306700), representing 85 to 90% of all hemophilia cases, is a deficiency of factor VIII, also known as antihemophilic factor. Factor VIII deficiency is the most common type of hemophilia and inherited as an X-linked recessive trait. Therefore, males are affected, females are carriers, and male-to-male transmission does not occur.

Hemophilia B or Christmas disease (OMIM number 306900) is caused by a deficiency of factor IX (plasma thromboplastin component) and also inherited as an X-linked recessive trait. Factor IX deficiency is one-fourth as prevalent as factor VIII deficiency.

Von Willebrand disease (vWD) (OMIM number 613130) is a hereditary bleeding disorder that results from an abnormality of the von Willebrand factor (vWF), which is found in plasma, platelets, megakaryocytes and endothelial cells. vWF circulates in conjunction with factor VIII and is important in platelet adhesion to the subendothelium via collagen and is, therefore, also important in the formation of the primary platelet plug. In von Willebrand disease, vWF may have a quantitative or qualitative abnormality based on the disease classification.

### Other Rare Hereditary Bleeding Disorders

Congenital deficiencies in coagulation factors other than factors VIII and IX are very rare. Factor XI deficiency in the Ashkenazi Jewish population has a prevalence of one in 1000; however, the prevalence of deficiencies of fibrinogen, prothrombin and factors V, VII, X and XIII are in the order of only one in 0.5 to 1 million.

Treatment for hemophilia involves intravenous replacement of factors VIII or IX using purified plasma-derived concentrates or, preferably, recombinant factor concentrates. The required dose, frequency and duration of therapy depends on the severity of the condition.^3^ For surgical procedures, patients receive preoperative doses of factor concentrate to raise plasma concentrations to hemostatic levels. These levels are then maintained postoperatively by repeated intermittent doses or continuous infusion.

To date, no universally standard protocol has been described to ensure safe oral and dental management for hemophilic children. Frequently, a fear of the bleeding risk in hemophilic children leads to the avoidance of dental care, which turns the child's need for simple, bleeding-free operative procedures into a need for risky extractions.

This report demonstrates the safe and successful delivery of full dental rehabilitation under general anesthesia with multiple extractions through efficient teamwork management and treatment options that minimized the risk of perioperative bleeding.

## CASE REPORT

A 7-year and 3-month-old known mild hemophilic child who was under the regular care of a hematologist and a pediatrician in a private hospital in Jeddah, Saudi Arabia, was referred to the dental center, complaining of pain and swellings of the gums.

### Medical History

The patient had no other medical disorders or history of surgeries. The patient had multiple incidents of nasal bleeding that required admission to the same hospital. The intraoral examination revealed the following:

- Missing teeth, numbers 51 and 52 due to normal shedding
- Decayed teeth, numbers 55, 74, 46 and 85
- Exposed teeth, numbers 65 and 75
- Badly decayed and infected teeth, numbers 54, 64 and 84
- Potential for crowding in the lower anterior segment

The treatment plan included the following:

- Composite resin restoration for teeth numbers 55, 74, 46 and 85
- Pulpotomy (ferric sulfate) and steel crowns for teeth numbers 65 and 75
- Extraction for teeth 54, 64 and 84
- Chairside ready-made space maintainers in place of teeth 54, 64 and 84
- Mesial slicing of the lower canines for temporary relief of crowding in the lower incisor region
- Sealant applications for teeth 16, 26 and 36
- Topical fluoride application.

### Child's Cooperation Level

On the Frankl behavior rating scale, the child's behavior and cooperation were rated as 2, corresponding to a negative behavior, manifested by a reluctance to accept treatment and uncooperativeness, with some evidence of a negative attitude that was not clearly pronounced.

A decision was made to perform the procedures under general anesthesia after considering the child's cooperation limits, the extensive dental procedures needed and the child's medical condition.

The child was admitted to the hospital 48 hours prior to the operation for factor VIII replacement therapy using the following formula: FVIII dose (IU/kg) = (desired increase in FVIII IU/dl) ü 2 to maintain an FVIII level of 70 IU/dl. This level was recommended by the hematologist based on the type of teeth to be removed (primary or permanent) and the anticipated amount of trauma that might be exerted during the dental procedures as stated by the pediatric dentist.

The preoperative blood investigation results were as follows:

- Bleeding time: 7 minutes
- Prothrombin time (PT): 13 seconds
- Thrombin time (TT): 31 seconds
- Activated partial thromboplastin time (APTT): 34 seconds.

The child's hematologist and pediatrician verified his fitness for dental work under general anesthesia, and an informed consent was signed by a parent.

Institutional ethics approval for publication was issued by concerned committees based on the institution's corporate social responsibility (CSR) standards. Informed consents were obtained from the parents and complied with Joint Commission for International Accreditation (JCIA) standards.

The child was advised and educated to perform gentle brushing with a soft toothbrush and to use a povidone iodine mouthwash after meals. The parents were instructed to stop meals for at least 6 hours prior to the time of surgery.

Preoperative complete blood counts showed low hemoglobin (10.6 g/dl); other values were within the normal range according to the patient's age group.

All procedures were performed according to the proposed treatment plan. The elapsed time during the dental procedures was 90 minutes. The child stayed for one hour in the recovery room and was sent to a ward for 48 hours for observation. The child was allowed to drink 2 hours following recovery and was placed on a cold, soft diet 4 hours after recovery.

### Anesthetic Considerations

After induction, intranasal intubation was used to avoid possible injury to the adenoids and subsequent bleeding. Furthermore, the tube was softened with warm water to help to minimize trauma during oral intubation.

During oral intubation, a mouth probe was inserted to help the anesthetist to fix the tube while the mouth was open. If the oral tube was inserted and fixed while the mandible was relaxed, it would have been hard for the dental operator to fully open the mouth and start the treatment.

**Fig. 1:**
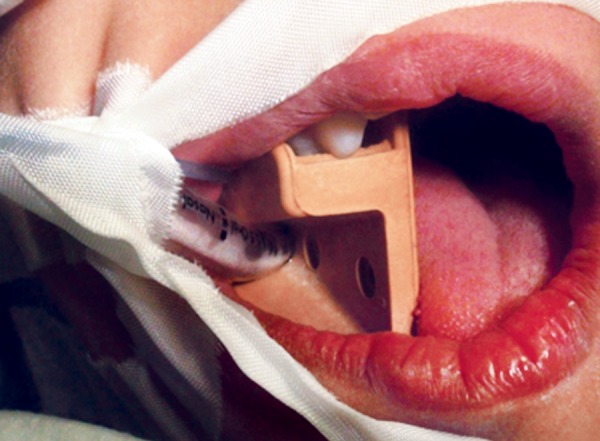
Tube accommodated inside the mouth gag to economize the use of the intraoral space

Oral intubation in a small child requires good intraoral space management for proper accessibility and vision. During the course of the dental treatment, the mouth probe was inserted in a reversed position to allow good retraction of the tube, which was accommodated inside the probe to economize the use of the intraoral space (Fig. 1).

As a routine, the author recommends the use of local anesthesia, even for patients undergoing treatment under general anesthesia, to minimize postoperative pain and aid patient homeostasis during surgery. The current case was not an exception to this routine. Nerve block injections were avoided to lower the risk of hematoma and/or internal bleeding.

### Positioning the Child

Children with hemophilia can suffer from internal bleeding of the joints and muscles should these tissues become strained. Adequate support for the knees and neck is routinely provided for all children undergoing dental care under general anesthesia, with special concern for hemophilic children. For the same reason, a mouth probe that would not cause excessive tension on the temporomandibular joint was selected.

### Moisture Control

**Fig. 2:**
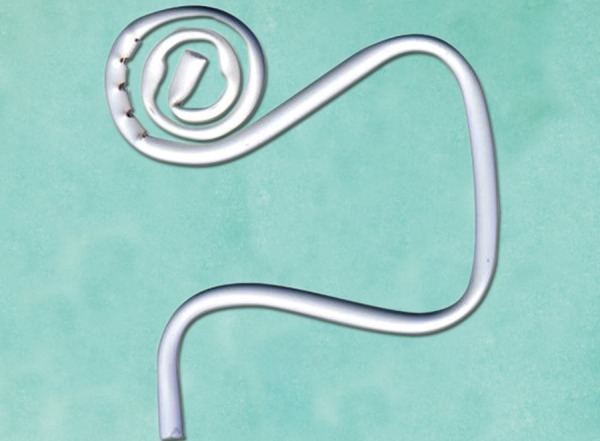
This suction tip minimizes trauma to the floor of the mouth and subsequent bleeding

Saliva ejectors are potential sources for intraoral trauma when firmly pushed into the sublingual mucosa, which increases bleeding risk in hemophilic children. Hygoformic saliva ejectors (Orsing Co.) were used to minimize possible trauma and subsequent bleeding risk (Fig. 2). The suction holes of these tips were directed away from mucosal tissues.

### Local Anesthesia

A few drops of a local anesthetic containing 2% lidocaine (Xylocaine, AstraZeneca Co) as a vasoconstrictor diluted 1:100,000 in epinephrine were used in the interdental papillae to help to control bleeding upon matrix band insertion for class II cavities. The same anesthetic was used intraligamentally prior to the dental extractions. Lidocaine (3%, Xylocaine, AstraZeneca Co) without epinephrine was used as intrapulpal anesthesia.^4^

### Extractions

**Fig. 3:**
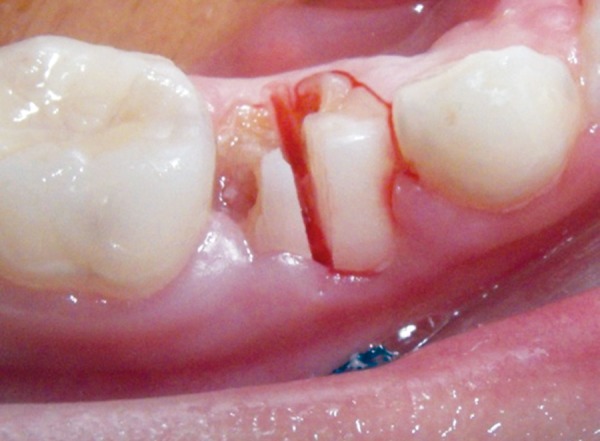
Root division and removal of the roots separately to minimize excessive dilatation of the socket

**Fig. 4:**
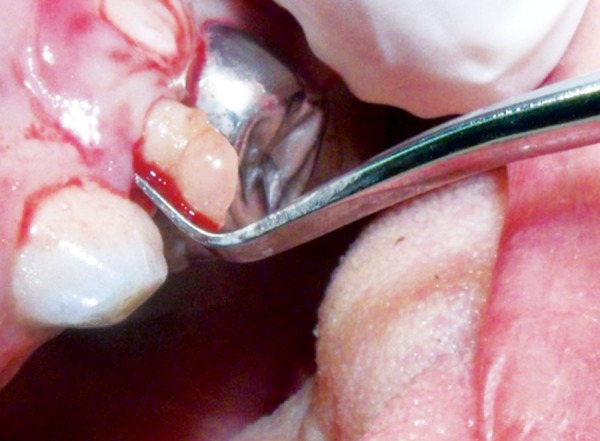
The use of plastic instruments reduces the traumatic force of extraction typically accompanying root elevator use

**Fig. 5:**
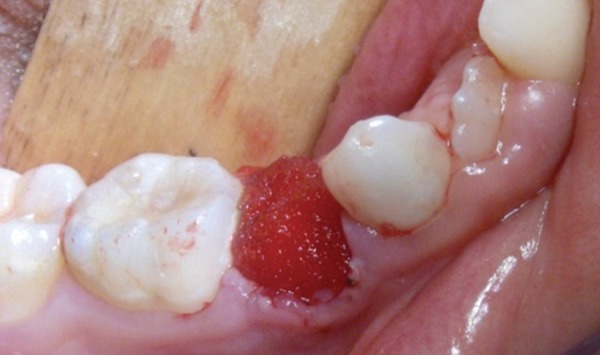
Insertion of a resorbable hemostatic foam inside the socket after extraction. The bleeding gingival collar was managed by using electrocautery

Avoiding unnecessary trauma was a primary concern. The following were considered:

1. Elective root division was used, and each root was extracted individually (Fig. 3).
2. Plastic instrumentation was used to remove broken down teeth instead of root elevators to minimize the use of excessive force (Fig. 4).
3. Extraction sockets can bleed through both gingival col tissue and alveolar bone; as such, gingival col bleeding was managed by using electrocautery, using caution not to produce sloughing tissue necrosis, which can further enhance bleeding.
4. Alveolar bone bleeding was managed using an absorbable surgical gelatin sponge (SurgisponÇ). Gelatin sponges have a porous structure that activates the thrombocytes at the moment blood contacts the matrix of the sponge. This causes the thrombocytes to release a series of substances that promote their aggregation concurrently with changes in surface character, thus enabling them to act as a catalyst for the formation of fibrin (Fig. 5).
5. Suturing material was prepared. An atraumatic suturing needle with 5-0 Vicryl Ethicon (Johnson & Johnson Co) was to be used for multiple adjacent extractions; the reported case did not present such a need.

### Pulpotomy Procedures

For the two teeth that required pulpotomy, ferric sulfate was used instead of formocresol. Ferric sulfate is reported to be a hemostatic agent with a comparable success rate to that of formocresol when used as a pulpotomy agent.^5^

### Stainless Steel Crowns

Preparation for steel crowns required subgingival reduction with subsequent bleeding; infiltration local anesthesia with a vasoconstrictor was utilized prior to tooth preparation to reduce the chances of excessive bleeding. The factory finish of the steel crown borders was left untouched. The use of scissors to adjust such borders was avoided to minimize the chance of postoperative mechanical trauma to the gingival tissues.

After crown cementation, excess cement was meticulously removed to ensure that remnants of hard cement material did not cause further mechanical irritation to the gingival tissues. The careful use of electrocautery aided in controlling bleeding after crown cementation.

### Restorative Procedures

Tooth restoration techniques involving sensitive materials, such as composites, which require proper surface management, acid etching, rinsing, drying and bonding, require a field that is clean of blood and saliva. Accordingly, restorative procedures were initiated prior to the extraction and steel crown procedures. Postponing blood contact procedures economized the use of time while the child as under general anesthesia and helped to provide an optimal environment for bonding and subsequent durability of the restorations.

### Postoperative Care

The patient was hospitalized for 48 hours, during which he continued factor VIII replacement as proposed by his hematologist and pediatrician. Oral amoxicillin and clavulanic acid postoperative antibiotics (457 mg/5 ml ) were administered every 12 hours for 7 days. For postoperative pain control, the preferred drug was acetaminophen 200 mg, administered every 8 hours for 3 days.

Dental health education regarding eating habits and food selection was provided to both the child and the parents. Gentle vertical brushing (the way the teeth grow) with a soft toothbrush was advised. The child was instructed to avoid vigorous horizontal scrubbing.

The postoperative recall schedule was weekly at first, followed by monthly and, eventually, every 6 months for 2 years. During postoperative recalls, topical Fluoridation, dental health education and check up for new caries lesions were performed.

Following the aforementioned postoperative recall schedule, no new carious lesions or pain was reported during two-year postoperative follow-up period.

## DISCUSSION

Fear of bleeding due to dental procedures in hemophilic children should not be a reason to avoid dental care.

Currently, the standard of care for patients with hemophilia is primary prophylaxis, involving regular infusions of factor VIII to make normal life possible.^6^Patients with hemophilia who enjoy adequate treatment have a life expectancy approaching that of the general population with a good health-related quality of life.^7^

The current case showed no family history of hemophilia, which might be attributed to the fact that up to one-third of hemophilia cases could be a result of factor VIII and IX mutations.^8^ Up to 30% of hemophilia A cases show inhibitor antibody development. However, in the case reported here, no factor VIII antibody development was reported by the hematologist and, accordingly, no immuno-suppressive agents were administered.

Weddlell and Jones in 1994.^9^ stated six situations that would justify the use of general anesthesia for delivering dental services.

1. Patients with certain physical, mental or medically compromising conditions.
2. Patients with dental restorative or surgical needs for whom local anesthesia is ineffective because of acute infection, anatomic variations or allergy.
3. Extremely uncooperative, fearful, anxious, physically resistant or uncommunicative children or adolescents with substantial dental needs for whom there is no expectation that the behavior will soon improve.
4. Patients with immediate comprehensive oral or dental needs who would otherwise not receive comprehensive dental care.
5. Patients requiring dental care for whom the use of general anesthesia may protect the developing psyche and/or reduce medical risks.
6. Patients who have sustained extensive orofacial and/or dental trauma.

The reported case fulfilled the first five situations. Treatment options were discussed in detail with the parents who agreed to have the procedure performed under general anesthesia. The aim of our treatment procedure, including fully restorative and preventive dental treatments, was to minimize the future dental needs of the child, therefore, minimizing the chances of bleeding associated with frequent therapeutic dental visits. Minimizing the patient's postoperative needs for treatment resulted in less stress during dental visits and allowed a trusting relationship to develop between the dentist and the child.

Dental health education was provided to the parents and their child. The topics were related to his medical condition, nutrition, dental needs and oral hygiene to promote general and dental health, and ensure that the child and his parents could provide satisfactory home care to a level that matched our treatment objectives.

A high percentage of Saudi parents do not comply with scheduled postoperative dental visits.^10^ In our case, we stressed the importance of complying with postoperative care to avoid the possible recurrence of caries and subsequent repetition of general anesthesia episodes.

Israels et al in 2006^1^ recommended the application of prefabricated vacuum-shaped splints on extraction sockets to help to control bleeding. In the reported case, this procedure would have necessitated the preoperative use of a stock tray, which can be traumatic, requires child cooperation and has a risk of unnecessary bleeding. Accordingly, a decision was made to proceed with the extractions, relying on the available bleeding control measures.

According to Powell and Bartle in 1974,^11^ the use of a rubber dam minimizes trauma to the gingival tissue, controls moisture and improves visibility. In the reported case, no rubber dam was inserted upon the request of the anesthetist for better visualization and monitoring of lip color and humidity inside the tube and to continuously ensure that the tube was in the proper place. The gingival seat for class-two restorations was carefully prepared without traumatizing the gingival tissue. A local anesthetic with a vasoconstrictor, as previously mentioned, was used to make the gingival tissue more resistant to bleeding during matrix and wedge insertions. The author highly recommends the use of magnifying dental loupes for all child patients; in this study, dental loupes proved very helpful in managing the fine details related to the prevention of operative and postoperative bleeding.

Rayen et al in 2011^12^ recommended indirect pulp capping for vital primary and permanent teeth for hemophilic children. As the objective of the treatment was to keep further dental needs and visits to a minimum, the author shifted treatment choices to those with more solid prognoses. Accordingly, pulpotomy was the treatment of choice for primary teeth with deep carious lesions.

Relapse and recurrence of caries are highly prevalent following dental treatment under general anesthesia. Foster et al in 2006 ^13^ reported that more than 50% of children who underwent total rehabilitation under general anesthesia developed new cavities during a 2-year follow-up period, with the majority of relapse cases composed of patients who did not fully comply with the routine follow-up schedule. In our case, the parents complied well with our proposed follow-up visits, dietary instructions and home care, which helped in keeping their child's further dental needs within preventive services and resulted in a better behavior management outcome.

## CONCLUSION

Dental management for a hemophilic child under general anesthesia is a multidisciplinary task that requires a team consisting of a hematologist, pediatrician, anesthetist and the pediatric dentist who acts as the coordinator of the team. The pediatric dentist is also responsible for the psychological management of the child patient and for providing proper dental health education to the child and his parents to gain the trust of the child and the cooperation of his/her parents. Parents are very important members of the team, managing the dental treatment for their hemophilic child. Their compliance with follow-up visits, dietary instructions and oral hygiene is essential in keeping their child's dental needs manageable within his/her cooperation margin. Full cooperation and understanding of each other's tasks and needs is must between each team member for successful dental management.

The author declares no conflicts of interest associated with the institution where the work was conducted or with the manufacturers and suppliers of the materials and medicaments described in our case report.
